# Electrical and thermal performance of bifacial photovoltaics under varying albedo conditions at temperate climate (UK)

**DOI:** 10.1016/j.heliyon.2024.e34147

**Published:** 2024-07-04

**Authors:** Aydan Garrod, Shanza Neda Hussain, Meet Hemantbhai Intwala, Amruthalakshmi Poudhar, S. Manikandan, Aritra Ghosh

**Affiliations:** a Faculty of Environment, Science and Economy (ESE), Renewable Energy Engineering, University of Exeter, Penryn, TR10 9FE, Cornwall, UK; b Department of Mechanical Engineering, Faculty of Engineering and Technology, SRM Institute of Science and Technology, Kattankulathur, Tamil Nadu, 603203, India

**Keywords:** bPV, Electrical, Bifaciality, Efficiency, Temperature

## Abstract

Bifacial photovoltaics (bPV) provide an advantage over their traditional monofacial counterparts as they can utilise solar radiation incident on both the front and rear side of the module, allowing for increased energy production. While this is an advantage on the side of bPV, the amount of energy produced by the rear side of the bPV panel can vary greatly depending on the inclination and azimuth of the panel as well as external climatic and ground albedo conditions. This paper aims to analyse the electrical and thermal performance of bPV under varying albedo conditions, using two different materials: grass, and a reflective material. It also contains an example of bifaciality testing under real-world conditions. Several experiments were conducted to evaluate the electrical performance and the temperature distribution of the bPV panel under different weather conditions at the UK location and then the results were compared with the single diode model of bPV. Furthermore, a bPV panel temperature model has been developed for the temperate climate condition of UK. The results show that the open circuit voltage of the system increases with irradiance up to around 800 $W/m^{2}$, at which point it decreased due to the increased PV system temperature. The normalised efficiency for the PV system under different conditions were also evaluated, which showed that the bPV module was most efficient under diffuse irradiance conditions, encouraging the use of the bPV technology in the UK.

**Table:** Nomenclature

A	Ampere	**Subscripts**	
DC	Direct Current	g	grass
DHI	Diffuse Horizontal Irradiation (W/m^2^)	gb	Bang gap
E	Energy	max	Maximum
F	View Factor	mp, mpp	Maximum power point
G	Irradiance (W/m^2^)	norm	Normalised
GHI	Global Horizontal Irradiation (W/m^2^)	oc	Open Circuit
I	Current (A)	p	Parallel
N	Number	ph	Photo/light
P	Power (W)	s	series
PV	Photovoltaics	sc	Short Circuit
R	Resistance (Ω)	sc,front	front side short circuit
Rb	Tilt Factor	sc,rear	rear side short circuit
STC	Standard Test Condition	ref	reference
T	Temperature (^o^C)	refl	reflector
V	Voltage (V)	t	Thermal
Greek Letters		total	Total
α	Altitude angle (degree)	N	Normalised
β	Slope (degree)	P	Panel
ρ	Reflectivity	POA	Plane of array
η	Efficiency (%)	SP	Shaded Portion
ϕ	Bifaciality factor	USP	Unshaded Portion
ψ	Temperature coefficient for current (A/^o^C)	Superscript	
μ	mean	f	Front
σ	Standard deviation	r	Rear
λ	Temperature coefficient for voltage (V/^o^C)		

## 1 Introduction

Global warming occurs due to a reliance on fossil fuels by economies across the globe [1]. It is necessary then for countries to diversify their sources of energy and adopt renewables whilst also ensuring that energy remains sustainable, accessible, and affordable [[2], [3], [4], [5], [6], [7]]. As of 2021, renewable energy generation stood at an all-time high of 30 % with advances to 50 % and 80 % expected by 2030 and 2050 respectively [8,9], but for the growth of renewables and to decrease reliance on fossil fuels and the emissions of greenhouse gases, levels of which in the decade 2010–2019 were higher than any level previously recorded [5,6], investment in green energy is crucial [4]. For many years now solar photovoltaics (PV) energy has been part of the solution, however, its energy density is lower than many other alternative energy sources [10]. Therefore, it is necessary to try and increase the energy density of solar PV where possible. This brings the introduction of bPV, the first examples of which showed up in the early 1980s [11,12]. The market share of bPV is expected to be around 70 % by 2030 [13] meaning that detailed knowledge of the performance of bPV systems is essential. bPV systems are able to produce more energy than their monofacial counterparts due to their ability to exploit irradiance incident on both the front and rear sides of the panel [14]. The advantage of bPV systems depends on specific site characteristics such as ground albedo [15]. When applied in a real-world situation, in a ground-mounted system, bPV systems can offer 25–30 % additional power output when installed in an optimised configuration [16].

In the process of exploring the bPV technology, several research has been carried out around the globe. In one such attempt in Canberra, Australia, it was found that bPV on a rooftop can result in an energy gain of up to 22.6 %, implying a considerable potential for optimizing solar energy production [17]. In another study carried out in Catania, Italy where the experimental setup was employed, it was observed that under operating conditions bPV exhibits more heat as compared to monofacial. This was estimated to be between 9 °C and 12 °C, depending on the season though there was a significant increase in the energy yield [18]. In South Korea, when monofacial and bPV modules were installed in a solar carport system and studied for a year, it was estimated that the total annual energy yield for bifacial was 3.08 % higher. The low yield was determined to be the attribution of the low bifaciality of the cell and the shading experienced [19,20]. As the technologies continue to develop and integrate with other sectors the employment of bifacial has proved to increase the electricity yield in the field like floating PV where modules can increase productivity due to reflection from water [[21], [22], [23]], and agrivoltaics by providing partial shading for shade-tolerant crops [24]. In a study performed, it was determined that using bifacial for vertically mounted agrivoltaics almost doubled the energy yield compared to monofacial [25]. In an experimental analysis, the performance of building integrated PV façade was examined, where the impact of ventilation on the temperature of the bPV and monofacial PV modules was investigated. It was observed that the module efficiency increased at the same time decreasing the thermal load, at the same time the temperature recorded on the back side of the bPV was higher as compared to its counterpart during the day [[26], [27], [28]].

However, much of the previous work done on bPV performance has been simulation studies [29,30] however, previous studies have measured the electrical performance of bPV modules for various applications such as building applied/integrated [31], agrivoltaic [[32], [33], [34]], floating [35,36], as well as a summary of several ground-mounted bPV simulations are mentioned in Ref. [37]. [38] evaluates bPV electrical performance, however, it focuses on indoor conditions and does not use conventional panel ground mounting [39]. Table 1 states previous bPV studies and their outcomes, focusing on ground-mounted systems. The UK currently has limited experimental papers evaluating bPV performance, and only one simulation-based study [40]. It is therefore vital that this growing technology be evaluated for a country whose solar industry is seen as a promising source of clean and affordable energy [41,42]. Given the UK's temperate climate [43] it could be thought that it would be a beneficial place to make use of bPV due to the technology making more use of its bifacial gain under irradiance conditions dominated by diffuse light [[44], [45], [46]]. The specific location of a PV system concerning its latitude, longitude, and typical climate will have a significant effect on its overall performance [47,48], this has the potential to be especially true with respect to climatic conditions when considering bPV systems, due to the different characteristics concerning temperature performance [49], and the earlier mentioned improved bifacial gain under diffuse light conditions.

**Table 1:** Previous bPV studies and their outcomes.

Paper	Location	System	Aims
[14]	Italy	Fixed tilt	Optimisation of bPV set up in ground mounted conditions
[24]	Indoor testing	Flash testing	Proposes methods for bPV characterisation
[36]	Jaipur, India	Single module, single axis tracked system	Evaluates effects of different shadings on the electrical performance of bifacial modules
[37]	Seoul, South Korea	Fixed tilt	Analysing the effect of shading compared to conventional monofacial modules
[50]	Edinburgh, Scotland	Fixed tilt	Evaluates bPV performance at different conditions compared to monofacial panels
[51]	Winterthur, Switzerland	Vertical, roof mounted	Evaluates performance of vertical bifacial performance on a green roof, comparing performance to a monofacial system and considering effect of roof vegetation
[52]	Le Bourget-du-Lac, France	BIPV facade	Evaluates the energy yield of a bPV façade as well as temperature performance
[15]	Europe, not specified	Roof mounted tilted	Bifacial and monofacial system comparison

As seen in Table 1, while there have been experimental studies on the performance of bPV systems, there is a relatively limited selection. None of which have been conducted in the United Kingdom, which as stated earlier could be an ideal location for the use of the bPV technology, due to its ability to perform better under diffuse dominant conditions, as shown later in this study. Also, the panel temperature model of the bPV system for the temperate climatic condition of United Kingdom is also not available. Based on these research gaps, the objectives were framed. The overall aims of the study are.

• Investigate the bifaciality of the bPV system in real-world temperate climatic conditions.
• To assess and compare the experimental and theoretical performance of the bPV system in different weather conditions by varying albedo.
• To assess the temperature distribution experimentally under varying weather conditions and develop a temperature model of the bPV system in the UK.
• To assess the effect that different climatic conditions, have on the electrical characteristics of the bPV system.

## 2 Methodology

### 2.1 Measurement equipment

Table 2 shows the equipment that was used for carrying out the tests outlined in this paper. Different measurement pieces of equipment were used to aid in different aspects of the test. A self-built test rig was used to mount the bPV module to be able to vary the tilt angle of the panel, using trigonometry to calculate the tilt angle. As well as this, measurement equipment was used, including an IV tracer, and pyranometer. T-type thermocouples were employed to measure the bPV cells' temperature, as well as a datalogger for use with the thermocouples.

**Table 2:** Measurement equipment used in the test.

Equipment	Purpose
PV test rig	Mount the bPV module off the ground and vary the tilt angle.
T-type thermocouples	Measuring cell temperature
Thermocouple datalogger	Logging cell temperature
IV-tracer	Measuring the electrical performance of the module
200R irradiance meter	Measuring front-side irradiance

For the measurement of irradiance at the surface of the solar panel SEAWARD-200R irradiance meter is used. To measure the voltage and current value SEAWARD-PV200 IV tracer is used. The device measures various parameters including open circuit voltage (V_oc_), short circuit current (I_sc_), maximum power point voltage (V_mpp_), and ground continuity. It also calculates the fill factor of the PV module/system.

To get accurate data of voltage and current with irradiance, SEAWARD-200R irradiance meter is connected to SEAWARD-PV200 IV tracer. Following are the technical specifications of the SEAWARD-PV200 IV tracer as mentioned in Table 3 [53]. Table 4 listed the essential parameters of the PV system used for the experiment.

**Table 3:** Parameters of the IV tracer used in the experiment.

SEAWARD-PV200	Display Range	Measuring Range	Accuracy
**Open Circuit Voltage Measurement**	0.0 V DC - 1000 V DC	5.0 VDC - 1000 V DC	± (0.5 % + 2 digits)
**Short Circuit Current Measurement**	0.0 ADC - 15.00 A DC	0.50 ADC - 15.00 A DC	± (1 % + 2 digits)
**DC operating Power**	0.0 kW–40 kW	0.50 kW–40 kW	± (5 % + 5 digits)

**Table 4:** Essential electrical parameters of the commercially available bPV system investigated in this work.

PV module parameter	Value from datasheet under STC
Rated power (W)	115
I_mpp_ (A)	6.12
V_mpp_ (V)	18.80
V_oc_ (V)	21.90
I_sc_ (A)	6.70
Efficiency	18.6 % [54]
Nominal Operating Cell Temperature (NOCT)	40 °C – 85 °C [54]
Temperature co-efficient of P_max_	−0.38 %/° C [54]
Temperature co-efficient of V_oc_	−0.28 %/° C [54]
Temperature co-efficient of I_sc_	0.06 %/° C [54]
Cell type	PERC - monocrystalline
Measured bifaciality factor (%)	45.60 %
Dimensions	786x763x30mm
height above ground	0.50 m
tilt angle	37°
Orientation	South facing
Grass albedo	0.21
Reflective material albedo	0.90

### 2.2 Bifaciality measurement

**Fig. 1:**
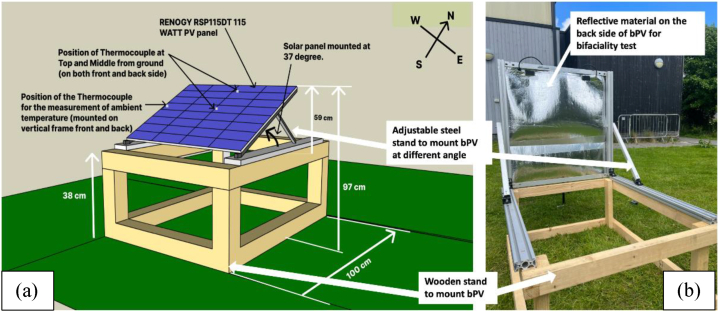
(a) Experimental setup for measurements. Renogy RSP115DT PV panel placed at 38 cm height from ground at a 37-degree angle facing south orientation. Height of the PV panel from the ground is 97 cm, (b) Bifaciality testing for front side performance while the back side was covered by a reflective film (University of Exeter, Penryn campus (50.16°, -5.12°).

A standard method for bifaciality testing is currently undefined [55]. The bPV module was placed on the wooden stand with dimensions 100 cm × 38 cm. On the top surface of the wooden stand, the bPV module was mounted on an adjustable steel stand at a 37° angle facing south direction. The bPV module was mounted at a 37° angle facing the south direction at 38 cm height as shown in Fig. 1a & Fig. 1b. To test bifaciality the panel was set at a 90° angle of incline to ensure an identical angle of incidence for both sides and one side of the bPV panel was covered to block irradiance to one side at a time, ensuring identical irradiance incident on exposed side measured by the 200R irradiance meter. The experimental setup for this procedure can be seen in Fig. 1a, with reflective material applied directly to the face of the side not being tested to ensure no light reaches it. I–V curves were generated for each side, and from that the short circuit current from the front and rear sides was used to calculate the bifaciality factor through Equation (1):

(1) $$Bifaciality\;factor=\frac{I_{sc,rear}}{I_{sc,front}}$$

**Fig. 2:**
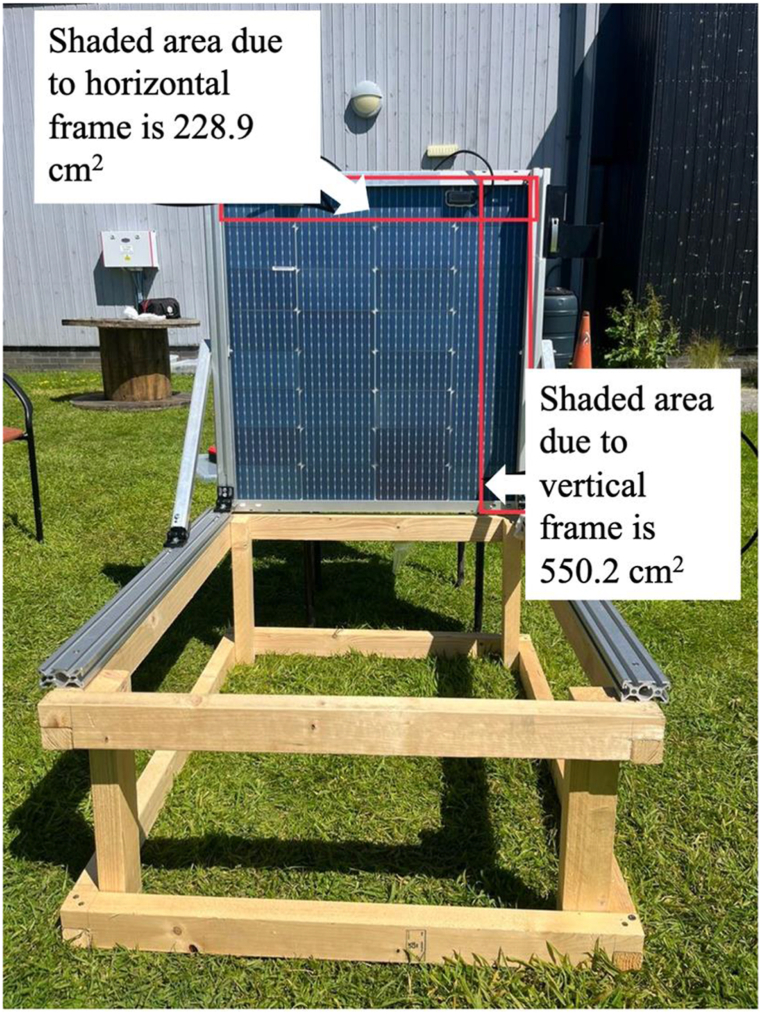
Effect of shading on the rear side of the panel affecting the outcome.(2)Totalshadedarea=(areashadedbytheshadowofhorizontalframe+areashadedbyshadowofverticalframe+2(areashadedbyjunctionbox)−(commonareashadedatthecornerbytheframe)$Total\;shaded\;area=\left(550.2+228.9+2\left(15.6\right)\right)-21=790.6{cm}^{2}$

For the bifaciality measurement, the rear side voltage and current data were taken by placing the bPV panel at 90° and at the same time the front side was covered with reflective material. For the rear side, the shaded area was 790.6 cm^2^ out of 5997.18 cm^2^ as shown in Fig. 2 using equation (2). During the time of reading approximately 13.18 % of the area of the rear side was shaded due to frame shadow. In this calculation, the shaded area by two junction boxes was also considered. The important parameters from bPV system during bifaciality experiment and illustrated in Table 5.

**Table 5:** Essential parameters from bPV system during bifaciality experiment (2023).

Side	V_oc_ (V)	I_sc_ (A)	Fill factor	P_mpp_ (W)	Irradiance level $\left(\frac{W}{m^{2}}\right)$
Front	20.7	5.92	76	93.0	910 ± 7
Rear	20.7	2.70	74	41.6	910 ± 7

**Fig. 3:**
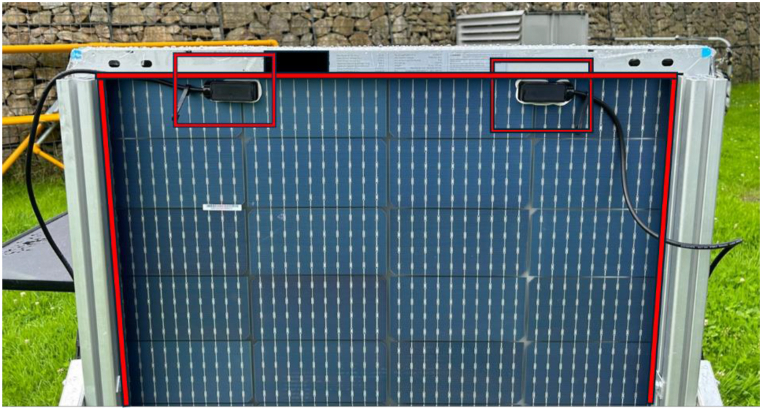
Photograph of the back side of the bPV module used for outdoor experiments at Penryn, (50.16°, -5.12°) August 15, 2023.

The measured bifaciality of 45.6 % seen from results in Table 4 is significantly lower than what was expected from this type of panel, as seen in examples mentioned in Ref. [37], however, this could be attributed to the lack of the ability to be able to flash test both sides of the panel, the approach described in the draft of IEC 60904 part 1–2 draft mentioned in Ref. [56], though this seems unlikely as the conditions on the day of testing were satisfactory with the irradiance at 90 % of STC conditions and clear skies. It could also be due to noticed bad practices in the production of the bPV module demonstrated in Fig. 3, highlighted in red.

**Fig. 4:**
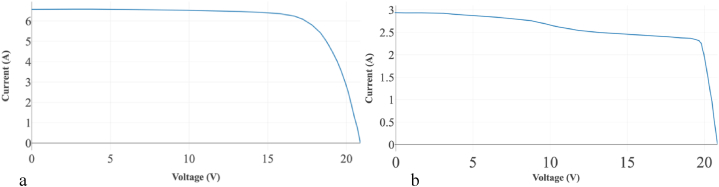
IV graphs of different sides of the bPV system. a) IV graph for the front side at 903 W/m^2^, August 15, 2023. b) IV graph for the rear side of the system at 917W/m^2^, August 15, 2023.

Several factors noticed on the backside of the panel could contribute to the low bifaciality factor, notably the frame encroaching over the panel contributing to self-shading of the cells on the outside of the module, and the two electrical connectors on the top left and right of the panel covering a portion of all the cells on the top row. Fig. 4a and Fig. b show the I–V graphs for this bPV system. The effect of shading shown in the previous figure on the rear side of the bPV panel can be seen in Fig. 4b with the noticeable change in gradient.

### 2.3 Experiment setup

**Fig. 5:**
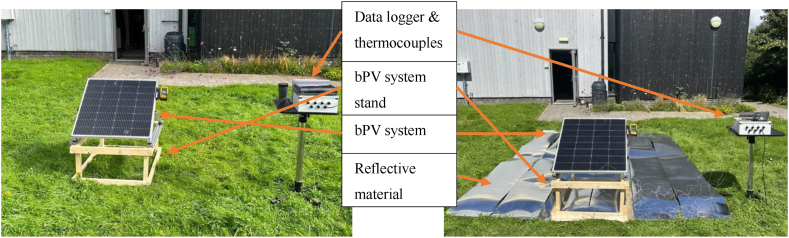
Experimental set-up to perform outdoor characterisation of a bPV system in UK climate at the University of Exeter campus.

The bPV module was mounted at an azimuth of 180° and a module tilt angle of 37°, the same that is used in Ref. [55]. Two ground reflectivity setups were used. One was grass, to simulate the albedo and conditions that most ground-mounted panels are mounted over, and the other material was a highly reflective foil in order to maximise the benefit of bifaciality. The experimental setup can be seen in Fig. 5.

### 2.4 Reflective material setup

**Fig. 6:**
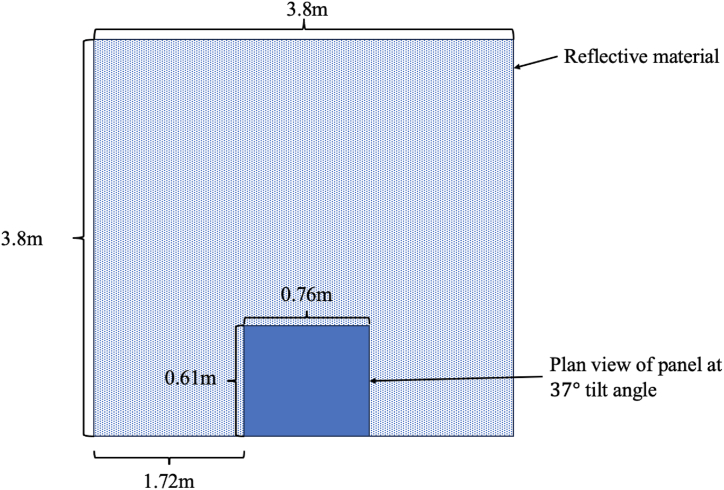
Plan view of the experimental set up when using the reflective material.

It was decided that the reflective material, should extend two times the module's width, and four times the module's length back in order to ensure that as much of the irradiance incident on the panel's rear surface had been reflected off of the foil as shown in Fig. 6. Increasing the albedo of the ground on which a panel is mounted has been proven to improve the bifacial gain of a system [50].

### 2.5 Model development and experimental validation

**Fig. 7:**
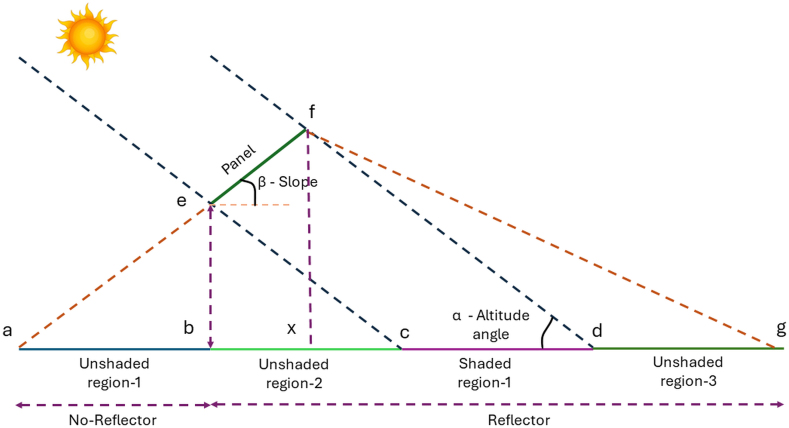
2D schematic of the bifacial PV system.

The data collected from the experiment for the bifacial panel with and without a reflector has been validated using the view factor model for rear side irradiance and the single diode model for bifacial PV performance, respectively. Fig. 7 shows the 2D schematic of the experimental setup of the bifacial PV system.

The solar radiation incident on the rear side depends on the radiation received from the sky dome, unshaded regions 1–3, and shaded regions 1. In this, the unshaded region 1 has grass cover with a reflectivity ($\rho_{g}$)of 0.21, and the unshaded region 2–3 and the shaded region have a diffuse reflector with a reflectivity ($\rho_{ref}$) of 0.91. The view factor between the shaded and unshaded regions has been evaluated using the crossed-string rule. The radiation incident on the front and rear sides is then evaluated using equations (3), (4)) [57].

(3) $$G_{P}^{f}=\left(GHI-DHI\right)\times {Rb}_{p}^{f}+DHI\times F_{P\to sky}^{f}+GHI\times \rho_{g}\times F_{P\to g}^{f}$$

(4) $$G_{P}^{r}=\left(GHI-DHI\right)\times {Rb}_{p}^{r}+DHI\times F_{sky\to P}^{r}+\sum_{1}^{x}\left[\begin{array}{c} GHI\times \rho_{g/refl}\times F_{P\to USPx}^{r}\\ +DHI\times \rho_{g/refl}\times F_{P\to SPx}^{r} \end{array}\right]$$

Where, $G_{P}^{f}$ ,and $G_{P}^{r}$ are the radiation incident on the front and rear side of the bifacial module.

Once the radiation incident on the front and rear sides of the bPV module is obtained, along with the measured module temperature and bifaciality factor of 45.60 %, the equations for the single diode model have been solved using a Python program as presented by Johnson and Manikandan [58].

The manufacturer data sheet of the PV panel provides the I–V characteristics, voltage at open circuit and maximum power, current at short circuit and maximum power condition for 1000 W/m^2^ and 25^o^C i.e., standard test conditions (STC). However, during operation in real time (non-STC), the panel temperature and solar radiation will be different, as will the current and voltage. Therefore, to evaluate the actual performance of PV panels under real-time operating conditions, a single diode model is employed [58].

**Fig. 8:**
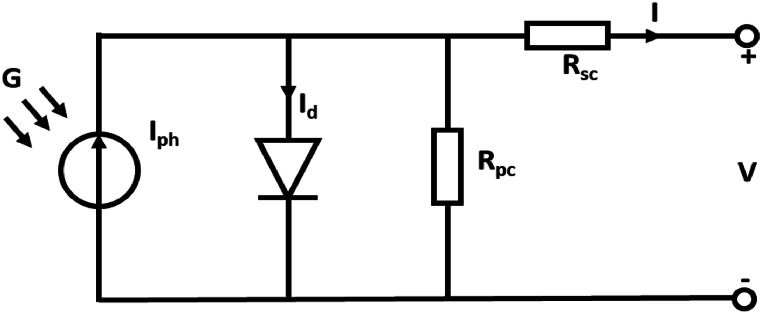
Single diode model of the PV cell.

The single-diode equivalent circuit of the solar cell is presented in Fig. 8. When a load resistance is connected to the terminal, the current flowing through the resistance can be evaluated as presented in equation (5). To solve this equation, the five parameters of the solar panel, which are thermal voltage, photocurrent, diode reverse saturation current, parallel resistance, and series resistance at STC conditions, are required. These five parameters can be evaluated using equations (6–10) presented in Table 6. Once these five parameters are obtained at STC conditions, a new I–V curve can be constructed for non-STC conditions by translating these STC parameters to non STC conditions using equations (11–15) presented in Table 7. Then, by substituting the five parameters at non-STC conditions in equation (5), the current and voltage can be obtained at non-STC conditions, thus obtaining the power output of the solar panel for the model.

(5) $$I=I_{ph}-I_{o}\left[\exp\left(\frac{V+I.R_{sc}}{N_{s}V_{t}}\right)-1\right]-\frac{V+R_{sc}I}{R_{pc}}$$

Where,

**Table 6:** I–V curve parameters extraction at STC conditions.

Sl.No	Parameter	Equation	
1.	Thermal voltage	$V_{t,ref}=\frac{\lambda T_{ref}-V_{oc,ref}}{\frac{N_{s}T_{ref}\psi }{I_{ph,ref}}-3N_{s}-\frac{E_{gb}N_{s}}{KT_{ref}}}$	(6)
2.	Photocurrent	$I_{ph,ref}\approx I_{sc,ref}$	(7)
3.	Diode reverse saturation current	$I_{o,ref}=I_{sc,ref}\;\exp\left(\frac{{-V}_{oc,ref}}{N_{s}V_{t,ref}}\right)$	(8)
4.	Parallel resistance	$R_{p,ref}=\frac{\left(V_{mp,ref}-I_{mp,ref}R_{s,ref}\right)\left(V_{mp,ref}-N_{s}V_{t,ref}\right)}{\left(V_{mp,ref}-I_{mp,ref}R_{s,ref}\right)\left(I_{sc,ref}-I_{mp,ref}\right)-N_{s}V_{t,ref}I_{mp,ref}}$	(9)
5.	Series resistance	$I_{mp,ref}=I_{ph,ref}-I_{o,ref}\left[\exp\left(\frac{V_{mp,ref}+I_{mp,ref}R_{s,ref}}{N_{s}V_{t,ref}}\right)-1\right]-\frac{\left(V_{mp,ref}+I_{mp,ref}R_{s,ref}\right)\left[\left(V_{mp,ref}-I_{mp,ref}R_{s,ref}\right)\left(I_{sc,ref}-I_{mp,ref}\right)-N_{s}V_{t,ref}I_{mp,ref}\right]}{\left(V_{mp,ref}-I_{mp,ref}R_{s,ref}\right)\left(V_{mp,ref}-N_{s}V_{t,ref}\right)}$	(10)

**Table 7:** I–V curve parameters extraction at non STC conditions.

Sl.No	Parameter	Equation	
1.	Thermal voltage	$V_{t}=\frac{T}{T_{ref}}V_{t,ref}$	(11)
2.	Photocurrent	$I_{ph}=\frac{G_{Total}}{G_{ref}}\left(I_{ph,ref}+\psi *\left(T-T_{ref}\right)\right)$ $G_{Total}=G_{f}+G_{r}\times \phi$	(12)
3.	Diode reverse saturation current	$I_{o}=I_{o,ref}{\left(\frac{T}{T_{ref}}\right)}^{3}\;\exp\left[\frac{qE_{gb}}{K}\left(\frac{1}{T_{ref}}-\frac{1}{T}\right)\right]$	(13)
4.	Parallel resistance	$R_{pc}=\frac{G_{ref}}{G_{Total}}R_{p,ref}$	(14)
5.	Series resistance	$R_{sc}=R_{s,ref}$	(15)

*I*_*ph*_ is Photocurrent/Light-generated current (A)

*I*_*o*_ is Reserve saturation current of the diode (A)

*R*_*sc*_ is Series resistance of the solar panel (Ω)

*R*_*pc*_ is parallel resistance of the solar panel (Ω)

*V*_*t*_ = *n·K·T*_*c*_*/q* (diode thermal voltage (V))

*n* is the diode ideality factor

*q* is the charge of the electron (1.602E−19C)

*K* is Boltzmann's constant (1.381E−23 J/K)

T_c_ is the temperature of the solar panel (K).

*N*_*s*_ is Number of solar cells in series in panel.

**Fig. 9:**
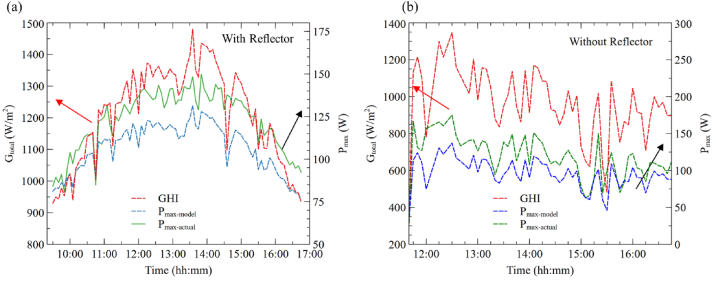
Comparison of actual and modeled power output of bifacial module (a) with reflector and (b) without reflector.

Fig. 9 presents the total solar radiation (rear plus front) as well as the actual and modeled power output. It is clear from Fig. 9 that the model has underestimated the actual power output, and the variation between the actual and modeled power output is −12.20 % and −11.28 %, respectively, for the with and without reflector cases. This is mainly because of the lower bifaciality factor of the module.

**Fig. 10:**
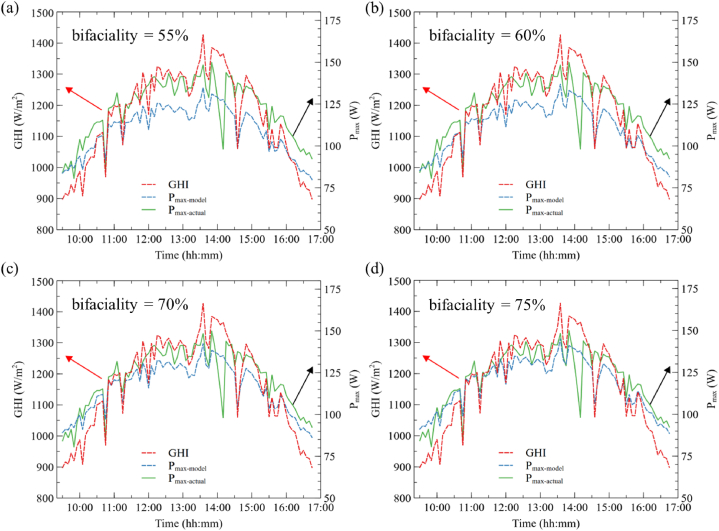
Comparison of actual and modeled power output of bifacial module for different bifaciality factor (a) 55 %, (b) 60 %, (c) 70 %, and (d) 75 %.

A study has been conducted with varying bifaciality from 55 % to 75 % using the single diode model, and the results are presented in Fig. 10. It shows that, with an increase in the bifaciality factor, the difference between the actual and modeled power output decreases. This is because, as the bifaciality factor increases, the power produced from the rear side also increases. The error percentage between the modeled and actual power out varies between −10.11 %, −8.20 %, −4.55 %, and −2.58 %, respectively, for the bifaciality factor of 55 %, 60 %, 70 %, and 75 %.

## 3 Results

**Fig. 11:**
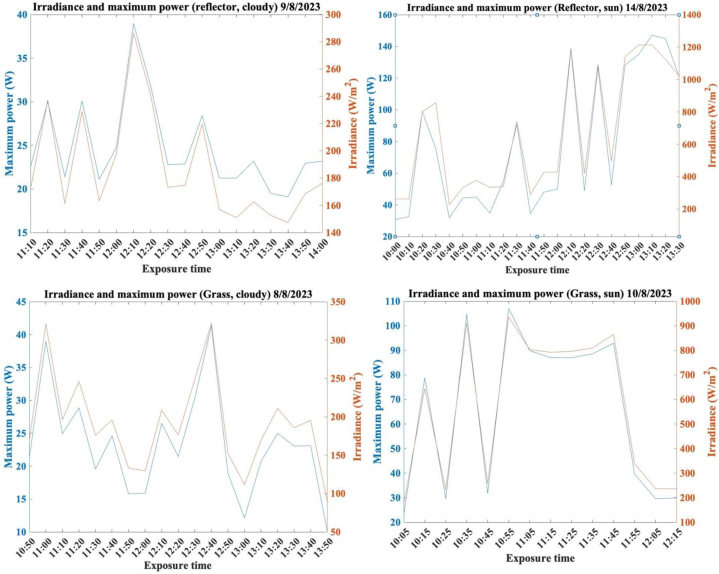
Power and Irradiance results for the duration of each of the experiments.

Fig. 11 shows the results for irradiance and maximum power produced by the system during the four different experiments. Readings were taken every 5 min, taking note of the irradiance, as well as the short circuit current, open circuit voltage, fill factor, and maximum power. Readings for the temperature of the different areas of the panel (front top, middle front, back top, middle back) were also taken using thermocouples, the results of which can be seen in section 3.3**.**

One noted feature of the results from the tests is that recorded results for P_mpp_, and I_sc_ were sometimes above the stated values for STC even when real-world conditions were less optimal than STC. This is not surprising as the field of bPV is relatively novel and still emerging, there is an unacceptable level of unknown testing parameters for the characterisation of bPV panels. The STC testing conditions for monofacial PV modules are well documented and described in IEC 60904. The STC performance stated on the datasheet of bPV modules as seen in this instance could be regarded as unreliable until a suitable standardised method of bPV characterisation becomes available. Something which should be done as soon as possible given bPV expected massive growth to dominate the PV market in the next decade [13].

### 3.1 Electrical performance analysis

#### 3.1.1 Normalised efficiency

Normalised efficiency is considered as equation (16) [59]. However, due to the novelty of bPV there is not an equation developed for bPV normalised efficiency. Nevertheless, this serves as a good reference to compare the normalised performance of the bPV module in different conditions [59]. It can also help show us the conditions in which bPV system is better able to exploit the bifaciality.

(16) $$\eta_{norm}=\frac{\left(\frac{P}{P_{STC}}\right)}{\left(\frac{G_{POA}}{G_{ref}}\right)}$$

**Fig. 12:**
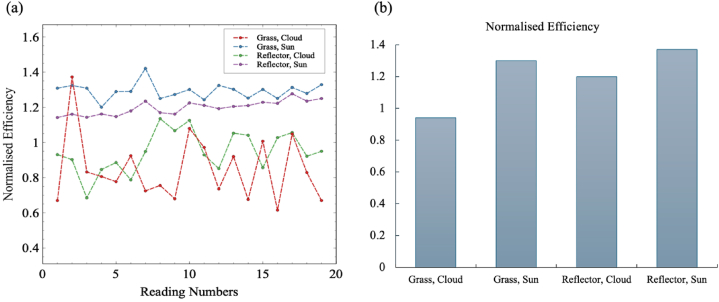
Normalised efficiency readings. a) Individual readings of normalised efficiency for different experiments. b) Average normalised efficiency value for each experiment.

Fig. 12 shows the normalised efficiency for the bPV panel under different albedo and climatic conditions. It can be seen that the normalised efficiency of the system is generally higher when the reflective material is used, showing the better ability of the bPV panel to exploit its bifaciality when there is more light being reflected off of the ground below it. However, it is also noticed that the normalised efficiency is largest for both reflector use and grass ground covering when there is total cloud cover, showing that the bPV panel is better able to exploit its bifaciality in conditions when the irradiance it receives is diffuse, and not majority direct like in the two sections of results with the lower normalised efficiency. It is also likely that the normalised efficiency for diffuse conditions would be higher if the system had a higher bifaciality factor.

For each experiments, standard deviation is calculated with the below mentioned equation (17).

(17) $$\sigma =\sqrt{\frac{1}{N}\;\sum_{i=1}^{Nu}{\left(Xi-\;\mu \right)}^{2}}$$

Where,

Nu = Total number reading for the experimental setup.

μ = Mean of the value.

σ = Standard deviation

*Xi* = Value of the reading of normalised efficiency.

From equation (17) standard deviation is calculated for normalised efficiency for different experimental setups. Standard deviation is 1.8 % and 6.6 % for experimental setup “Grass, cloud” and “Grass, sun” respectively. For reflective material cloud and sun setups standard deviation is 11 % and 28 % respectively. Standard deviation is higher in case of Reflector, sun is due to length of time duration of the material. Experimental setup “reflector, sun” is performed from 09:00 a.m. to 07:30 p.m. for nine and half hours. Due to the difference of power generation in the morning and afternoon standard deviation is higher.

#### 3.1.2 Open circuit voltage (V_oc_) performance

**Fig. 13:**
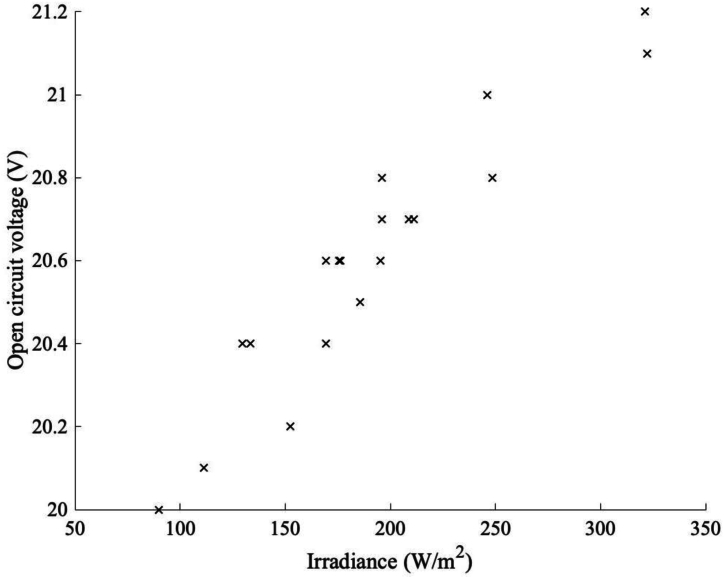
Relationship between Irradiance and open circuit voltage with a grass ground covering on a cloudy day.

It is known that the voltage of a solar cell will generally decrease as the temperature of the cell increases [60]. However, in temperate conditions such as the UK the climate conditions mean that the climatic temperature is often not high enough to start significantly affecting the performance of the PV module, as seen in this study. In fact, on days when there was thick cloud cover, it could be seen that when the irradiance increased, so did the voltage, as seen in Fig. 13.

**Fig. 14:**
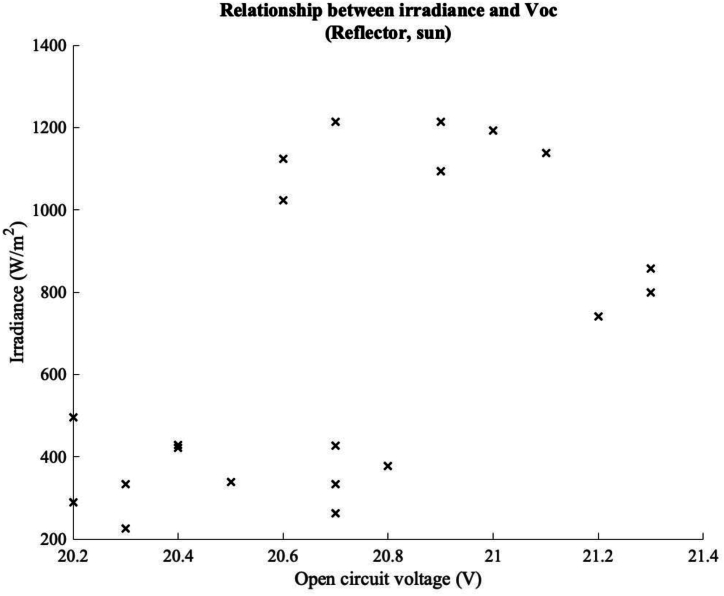
Relationship between Irradiance and open circuit voltage with a reflective ground covering and sunny conditions.

The relationship becomes more complicated however, when experiments were carried out in sunny conditions, Fig. 14 shows the relationship between irradiance and open circuit voltage on a sunny day using the reflectors. It can be seen that V_oc_ rises with irradiance up until around the point that the irradiance reaches $1000\;W/m^{2}$, at which point the temperature of the module starts to rise to a point that the output is negatively affected.

### 3.2 Thermal performance analysis

**Fig. 15:**
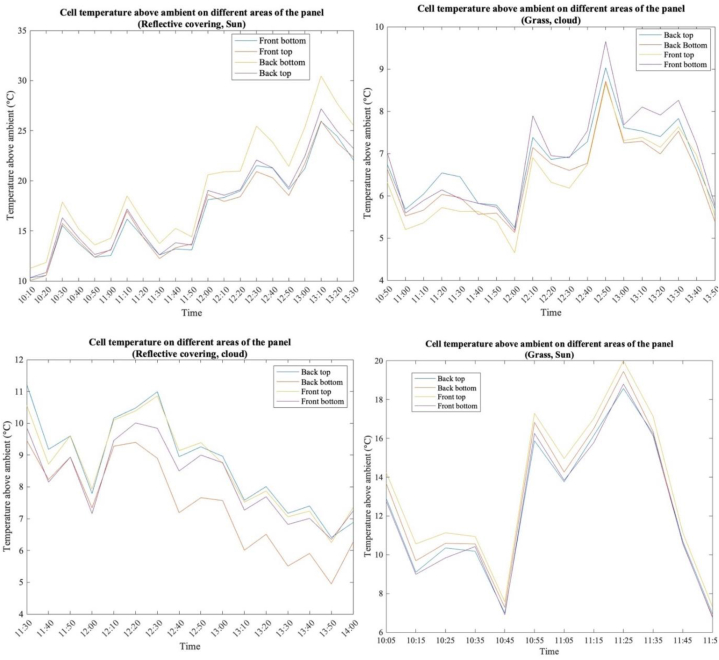
Temperature at different areas of the PV system under varying weather conditions.

The temperature of a PV module changes with irradiance [49,61], however, in a PV system it is not just irradiance that will contribute to the module temperature, the presence of plants can help reduce PV module temperature [62]. The use of grass, as well as a reflective material in the same climatic conditions, can help in understanding the thermal performance of bPV modules. For this study, four thermocouples were used on different areas of the bPV module to monitor the temperature change under different conditions. Thermocouples were placed on the top and bottom of the front and back of the bPV module. Significant changes were observed in the temperature distribution under different conditions, as can be seen in Fig. 15.

It should be noted that the PV system was in a location in which it was significantly sheltered from southerly and westerly wind directions. By considering this, variations in the temperature can be explained. On the days of testing the wind direction can be seen in Table 8.

**Table 8:** Wind direction on test days.

Conditions	Grass, cloudy	Grass, sun, and cloud	Reflective, cloudy	Reflective, sun and cloud
**Wind direction**	westerly	southerly	westerly	Southerly

On both of the days that the wind was blowing in a northerly southerly direction, the temperature at the bottom of the backside of the panel, which is the most sheltered part of the panel, with the wind also sheltered from these directions it can also be seen that the module temperature is significantly higher than the ambient temperatures than on days when the wind is not as sheltered however, on these days there was also significantly more irradiance which played a part in raising the modules temperature [63].

When the bPV panel was mounted over grass, the temperature in different areas of the module did not generally vary by any more than 2°C, however, this changed dramatically when the panel was mounted over the reflective material, with the temperature variation in the panel differing by up to 5°C, this was especially evident when the reflector was used in sunny conditions. Other noticeable trends in the data are the bottom of the front side of the panel was consistently at a higher temperature compared to other areas in overcast conditions, and at a lower temperature in sunny conditions. A main trend noticed especially when the reflective material was used in cloudy conditions, the areas of the panel that was the coolest, which was the rear side, was the hottest in sunny conditions, which may be due to the extra irradiance reflected onto the rear side of the panel and the reduced ability for ventilation due to the sheltering of the rear side of the panel from the frame and the mounting structure.

**Fig. 16:**
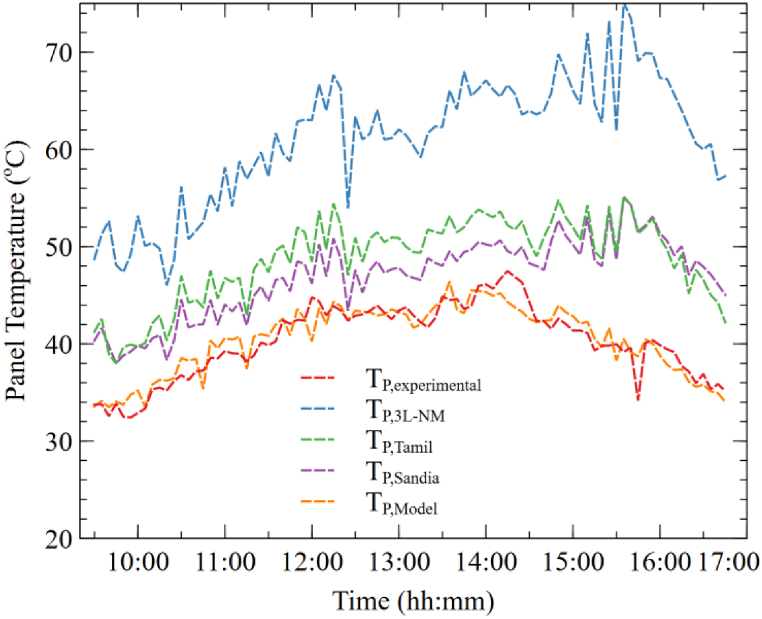
Predicted and experimental panel temperature of bifacial module.

The temperature of the bifacial module has been experimentally measured, and based on that data, a temperature model has been developed using multivariate regression with *G*_*total*_, ambient temperature, and wind speed as the variables. The total radiation is used instead of GHI, because the temperature of the bifacial panel depends on the radiation incident on the front and rear side of bPV. The modeled temperature, along with the experimental 3L-NM model [64], Sandia model [65], and Tamizhmani model [66], has been compared in Fig. 16, and most of the models have overestimated the panel temperature. Equation (18) displays the obtained equation for the temperature model from our study.

(18) $$T_{P,Model}=8.8862+\left(0.0231xG_{total}\right)+\left(0.1399xT_{a}\right)-\left(0.2137xWs\right)$$

Equation (18) implies that with an increase in total solar radiation and ambient temperature, the panel temperature will increase, but with an increase in wind speed (*Ws*), the panel temperature decreases because of increased heat transfer from the panel to the ambient. The modeled panel temperature has an R^2^ value of 0.834, and therefore, the new model can predict the bifacial panel temperature with the least error compared to other models.

**Fig. 17:**
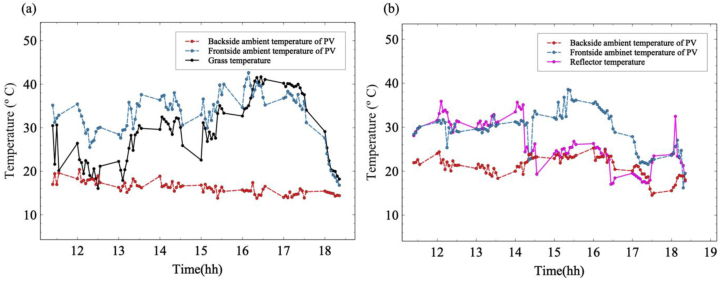
Ambient temperature for backside and front side of the PV system. (a) Backside and front side ambient temperature with temperature of grass for “Grass, Sun”. (b) Backside and front side ambient temperature with temperature of reflective material for “Reflector, Sun”.

Ambient temperature, temperature of the grass and reflective material played an important role during the experiment. Fig. 17 illustrates the various temperatures and the comparison between them. It is evident that the use of reflective material increased the temperature of the module resulting in an increase in the ambient temperature near the module for both front and rear sides when compared to the setup without the reflective material. This is because reflective materials increase the surrounding temperature by reflecting the solar radiation back in the opposite direction, the orientation of the material placed also plays an important role. When applied on a large scale, they can modify the ambient temperature, which can be observed, since the ratio of the area of the module to the reflective material in this experiment is 31.14. Similar outcome was observed in the work done by Ref. [67]. The reflectance of grass is much lower as compared to the reflective material and the albedo ranges between 10 and 25 % [68,69]. Due to the high reflection and absorption of the reflective material the change in the temperature has a direct relation between the received irradiances. During the hours of the day when the solar radiation is high the reflective material has higher temperature with the decrease in the intensity of radiation during the second half of the day the temperature falls. The heat retention property of the reflective material and the grass is also responsible for the variation depicted in the graphs. The grass is responsible for reducing the near-surface temperature significantly due to its evapotranspiration property thus resulting in the grass curve being mostly lower than the ambient [70]. Due to the significant radiation absorption coefficient of grass, a remarkable absorption of heat radiation takes place accounting for the cooling effect [71]. It is also interesting to note that the temperature for the initial 3–4 h for the grass was low, this is due to the presence of dew on the grass. Then there was a spike in the temperature of the grass as the dew evaporated completely and the moisture in the air near the ground has reduced, increasing the temperature, contrary to the employment of reflective material which almost maintained 30 °C–36 °C during the same period. However, the initial temperature is 30 °C for both setups. A drastic dip in the temperature was observed after around 14:00 h for the reflective material after which the temperature remained below its peak due to reduction in the solar irradiance. However, in the case of grass, the peak is reached between 16:00 and 17:00 when the grass temperature was seen to be higher than the ambient temperature for the front side of the module due to the heat retention of the grass. Though the back ambient temperature remained the lowest for both scenarios because of lack of direct radiation reaching the area, it was still higher when the reflective material was used compared to grass, and in some instances, it was slightly higher than the reflective material between 16:00 h and 17:00 h. The front side ambient followed mostly a similar curve pattern as the grass and reflective material though in the case of grass it was higher than the grass temperature until 16:00 h, on the contrary, it was mostly higher than the reflective material after 14:00 h.

## 4 Conclusions

This paper has analysed multiple performance factors of a bPV system under different climatic conditions, as well as varying the type of surface over which the system was mounted. The system was tested in both sunny and cloudy conditions, as well as mounted over grass, and a highly reflective surface. Several results were concluded from the tests. First of all, a method for testing the bifaciality of the panel was tested in order to conclude the ratio of efficiency of the front side of the system to the rear side of the system.

A finding from the experiments was the normalised efficiency of the system under different conditions, finding that the normalised efficiency was lowest under climatic conditions dominated by direct irradiance and that for conditions which were dominated by diffuse light for both ground coverings of grass and the reflective material though the difference in the normalised efficiency between conditions was greatest for the grass ground covering. This result is promising for the UK, as it has a temperate climate, and therefore has a large number of days where the weather is dominated by overcast conditions. This means that bPV could be extremely useful in helping the UK increase its production of electricity generated by renewable sources.

A second finding from the experiments was the open circuit voltage performance of the system. While the climatic conditions were dominated by diffuse light, while the levels of irradiance increased, so too did the open circuit voltage. However, if there were high levels of irradiance the effect of temperature increase appeared to have an effect, as the levels of irradiance exceeded 800 W/m^2, leading to a decrease in the open circuit voltage of the system. Finally, the temperature distribution of the system was investigated under different climatic conditions, and with the different ground coverings. It was noticed that the variation of temperature distribution on the system varied with ground covering and climatic conditions. Also, a panel temperature model has been developed to predict the bifacial PV temperature for the temperate climatic condition of UK.

## CRediT authorship contribution statement

**Aydan Garrod:** Writing – review & editing, Writing – original draft, Visualization, Validation, Resources, Methodology, Investigation, Formal analysis, Data curation, Conceptualization. **Shanza Neda Hussain:** Data curation, Formal analysis, Investigation, Resources, Validation, Visualization, Writing – review & editing. **Meet Hemantbhai Intwala:** Data curation, Formal analysis, Investigation, Resources, Validation, Visualization, Writing – review & editing. **Amruthalakshmi Poudhar:** Data curation, Formal analysis, Investigation, Resources, Validation, Visualization, Writing – review & editing. **S. Manikandan:** Data curation, Formal analysis, Investigation, Methodology, Resources, Software, Validation, Visualization, Writing – review & editing. **Aritra Ghosh:** Writing – review & editing, Visualization, Validation, Supervision, Software, Resources, Project administration, Methodology, Investigation, Funding acquisition, Formal analysis, Data curation, Conceptualization, Writing – original draft.

## Declaration of competing interest

The authors declare that they have no known competing financial interests or personal relationships that could have appeared to influence the work reported in this paper.
